# Supplementary material for: scPair: Boosting single cell multimodal analysis by leveraging implicit feature selection and single cell atlases
Source: Nat Commun. 2024 Nov 15;15:9932. doi: 10.1038/s41467-024-53971-2 (PMC11568318; doi:10.1038/s41467-024-53971-2)
Supplement: Supplementary file 1 — Supplementary Information [file 41467_2024_53971_MOESM1_ESM.pdf]

scPair: boosting single cell multimodal analysis by leveraging implicit feature selection and single cell atlases

Hongru Hu[1,2,*] & Gerald Quon[2,3,*]

[1]Integrative Genetics and Genomics Graduate Group, University of California, Davis CA

[3]Genome Center, University of California, Davis CA

[2]Department of Molecular and Cellular Biology, University of California, Davis CA

*To whom correspondence should be addressed: hrhu@ucdavis.edu, gquon@ucdavis.edu

List of Figures

Figure S1 2

Figure S2 4

Figure S3 6

Figure S4 7

Figure S5 8

Figure S6 10

Figure S7 11

Figure S8 12

Figure S9 13

Figure S10 14

Supplementary Figure:

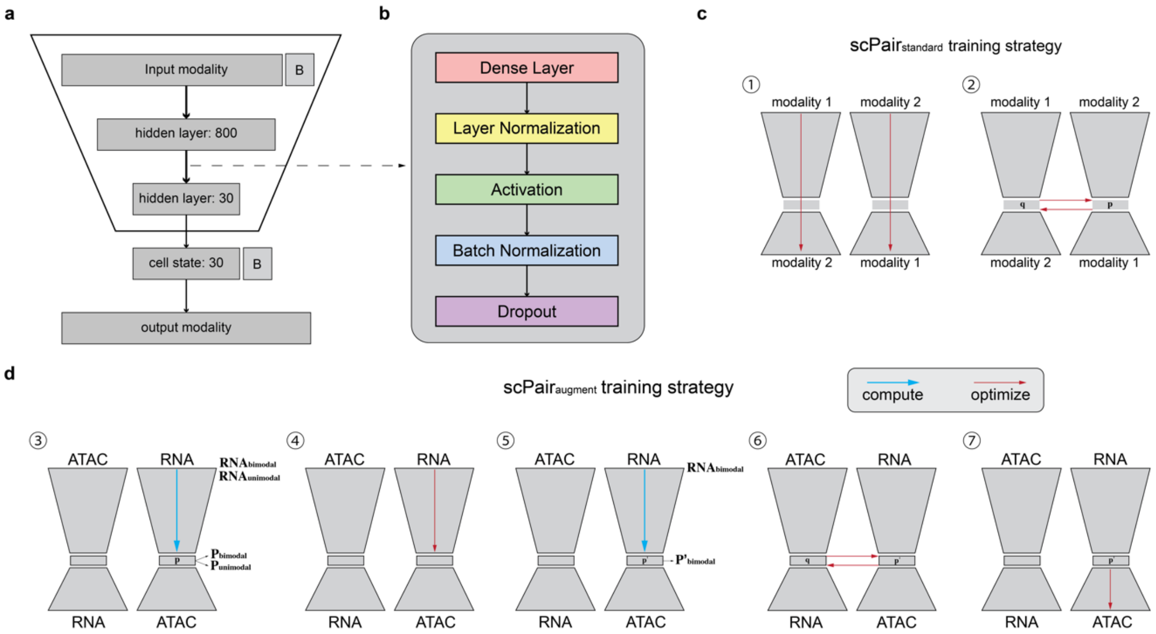

Figure S1

Training strategy for both scPair$_{standard}$ and scPair$_{augment}$. (**a**) Schematic illustrating the default setting of each modality-specific feedforward network. (**b**) The structure of a single layer of scPair. (**c**) scPair$_{standard}$ is trained using multimodal data only. (1) The feedforward networks are each individually trained with each data modality, separately. (2) The bidirectional mapping networks are then trained, given fixed parameters for the feedforward networks. (**d**) The scPair$_{augment}$ model starts after training scPair$_{standard}$. Assuming we have trained the scPair$_{standard}$ model using paired RNA and ATAC modalities, and we have available a unimodal scRNA-seq dataset, we first re-train the RNA encoder using both the unimodal scRNA-seq and the RNA component of the multimodal data used to train scPair$_{standard}$ in steps 1-2. To do so, (3) we compute the cell states (denoted as P) of the scRNA-seq and multimodal data using scPair$_{standard}$. (4) These input-output pairs then form the training data for re-training the RNA encoders, with mean-squared-error being minimized as a loss function. (5) Afterwards, we compute the updated RNA cell state (denoted as P’) using the RNA component from the multimodal data. (6) The bidirectional mapping networks are then updated. (7) Finally, the decoder predicting ATAC data from RNA cell state space is updated using the multimodal data. Blue and red arrows in (c) and (d) represent computations using the trained (fixed parameter) networks and optimization of parameters of the networks, respectively. Cell states P (from

scPair$_{standard}$) or P’ (from scPair$_{augment}$) derived from step (3) or (5) can be utilized for downstream tasks such as cell clustering and trajectory analysis.

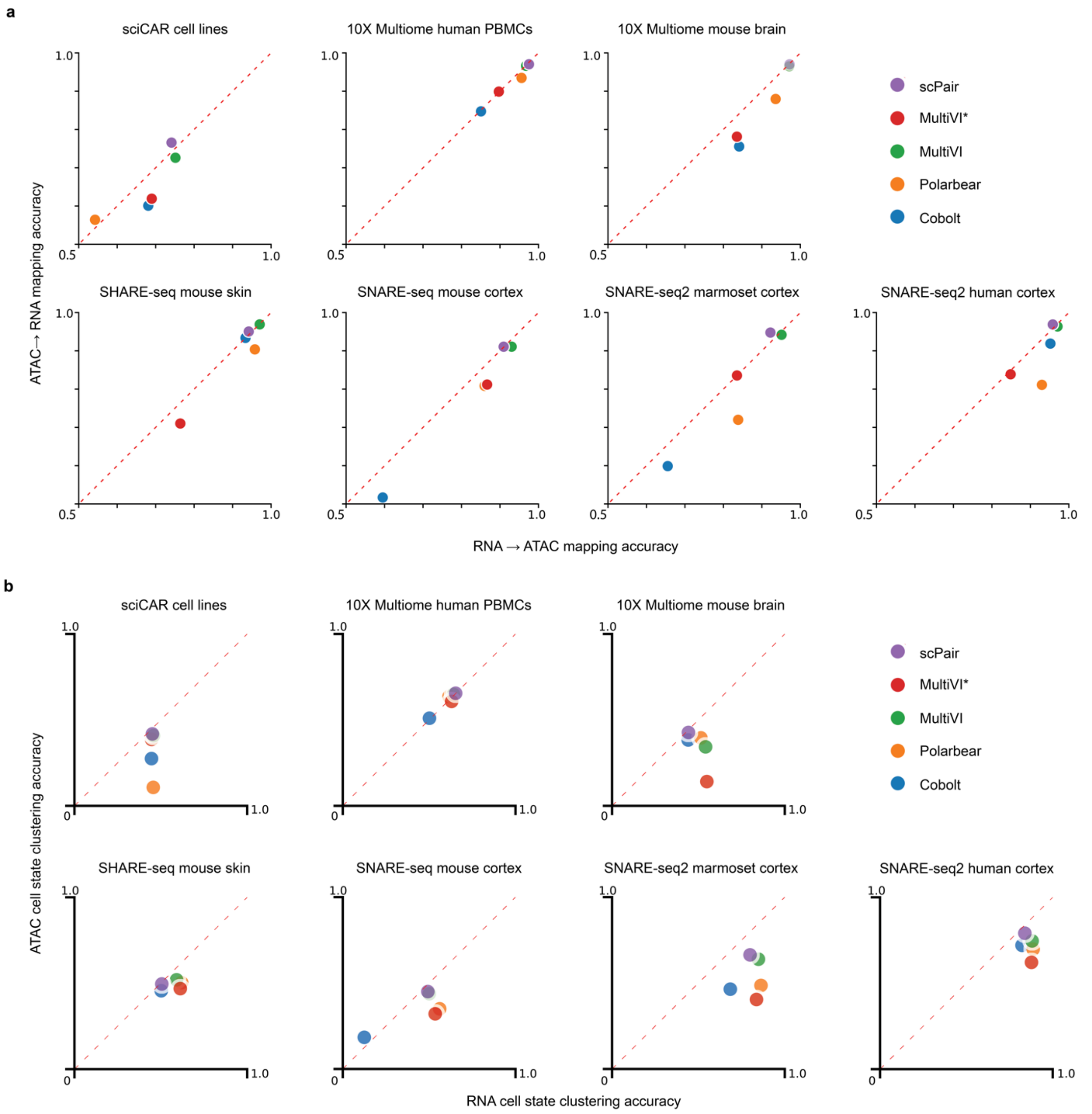

Figure S2

With the same training and held-out data sets from each study[1–5], scatter plots quantitatively compare the performance of **(a)** cross-modality mapping and **(b)** modality-specific clustering achieved by different methods[6–10]. **(a)** The y-axis measures performance using the Fraction Of Samples Closer Than the True Match (1-FOSCTTM) for mapping held-out scRNA-seq data to scATAC-seq, while the x-axis shows 1-FOSCTTM for mapping in the reverse direction. Points closer to the y=x line indicate more consistent bidirectional mapping, with points towards the top right demonstrating more accurate mapping in both directions. **(b)** The y-axis and x-axis measure accuracy using Normalized Mutual Information to compare the clustering accuracy on

the held-out data using different benchmarked methods, given the learned ATAC and RNA cell states, respectively. Points closer to the y=x line indicate more consistent clustering between modality, with points towards the top right demonstrating more accurate clustering in both modalities. Points are mostly below the y=x line, indicating those methods have systematically lower ATAC clustering accuracy, while scPair is closer to the line, indicating scPair clusters RNA and ATAC approximately equally. Source data are provided as a Source Data file.

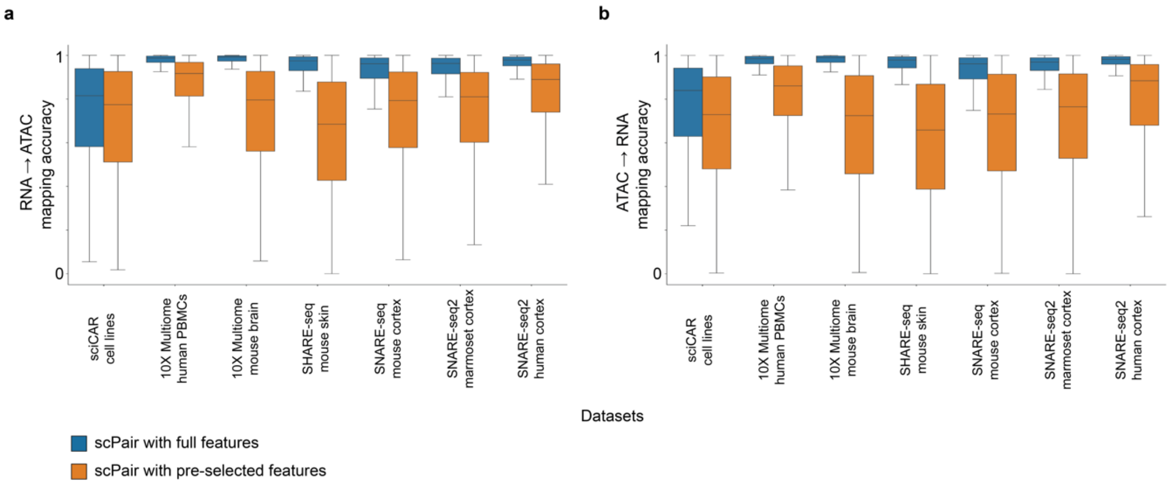

Figure S3

(a) Benchmark of RNA→ATAC mapping performance using scPair with no feature pre-selection and with feature pre-selection (blue and orange, respectively). Both strategies were evaluated using identical training and held-out datasets. Box plots display the mapping performance measured by the Fraction Of Samples Closer Than the True Match metric (1-FOSCTTM), where higher values indicate better performance. **(b)** Same benchmark as in (a), but for ATAC→RNA mapping performance of all methods. In the box plots, the minima, maxima, centerline, bounds of box, and whiskers represent the minimum value in the data, maximum, median, upper and lower quartiles, and 1.5x interquartile range, respectively. Source data are provided as a Source Data file.

a

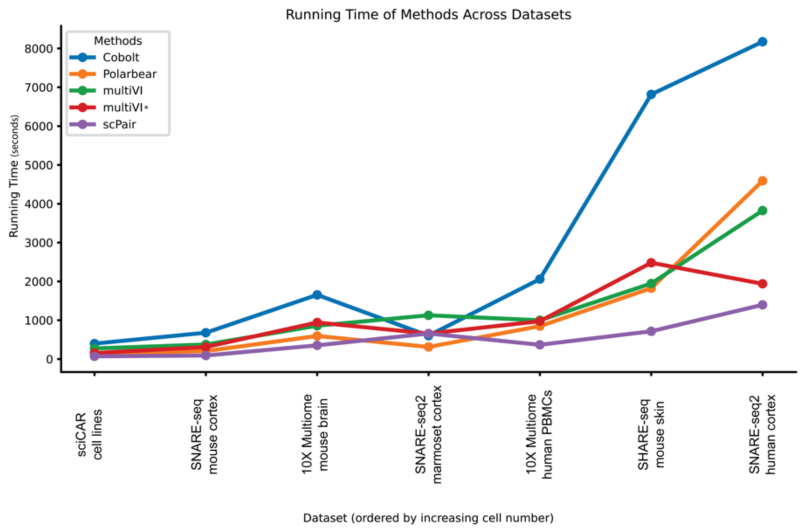

b

	number of cells	number of RNA features	number of ATAC features	RNA non-zero counts pct.	ATAC non-zero counts pct.
sciCAR cell lines	4,216	17,368	57,425	8.43%	0.55%
SNARE-seq mouse cortex	8,055	9,104	37,030	10.17%	3.52%
10X Multiome mouse brain	9,370	14,461	82,474	27.33%	5.76%
SNARE-seq2 marmoset cortex	9,946	20,292	35,573	18.85%	2.25%
10X Multiome human PBMCs	11,331	13,515	86,002	14.60%	8.09%
SHARE-seq mouse skin	34,774	15,436	97,669	4.15%	3.15%
SNARE-seq2 human cortex	84,178	16,139	33,517	13.67%	2.03%

Figure S4

(a) Benchmark of running times (in seconds) across datasets with varying numbers of cells, ranging from 4,216 to 84,178. Different colors represent different methods, all tested on a single GPU (NVDIA A100, 80GB). **(b)** Description of each benchmarked dataset, including the number of cells, the number of RNA modality features (genes), the number of ATAC modality features (peaks), and the percentage of non-zero counts in both modalities. The order of datasets (top to bottom) is the same as that in (a) (left to right). Source data are provided as a Source Data file.

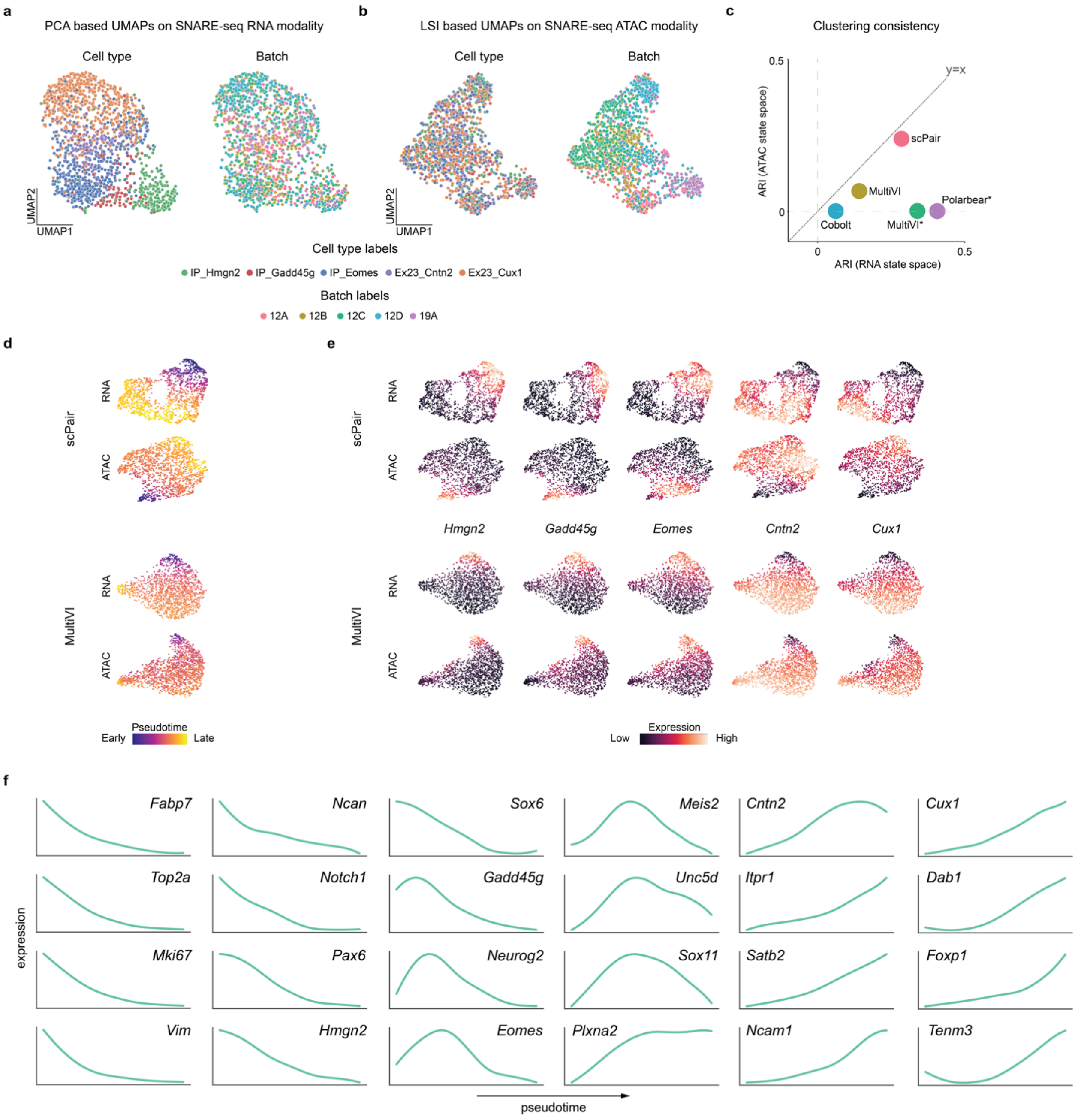

Figure S5

(a) UMAP visualizations of RNA principal components (PCs) using Seurat[11,12] on the neonatal mouse cortex SNARE-seq data, colored by cell types (left) and batch labels (right). **(b)** UMAP visualizations of ATAC latent semantic indexing (LSI) using Signac[13] on the neonatal mouse cortex SNARE-seq data, colored by cell types (left) and batch labels (right). (**c**) Scatter plot illustrates the consistency in clustering accuracy between the learned RNA and ATAC cell state spaces. Cell type clustering accuracy is evaluated based on the Adjusted Rand Index (ARI) metric (**Methods**). Points closer to the y=x line indicate higher consistency, with points toward

the top right demonstrating more accurate clustering in both modality-specific cell state spaces. **(d)** UMAP visualizations of modality-specific cell state spaces learned by scPair (top) and MultiVI (bottom), colored by the inferred pseudotimes that were estimated using the learned cell states and further processed by Palantir[14]. **(e)** UMAP visualizations of modality-specific cell state spaces learned by scPair (top) and MultiVI (bottom), colored by the expression levels of five cell-type-specific marker genes. **(f)** Line plots indicate the imputed RNA expression of the developmental stage-related marker genes[4] along the pseudotime.

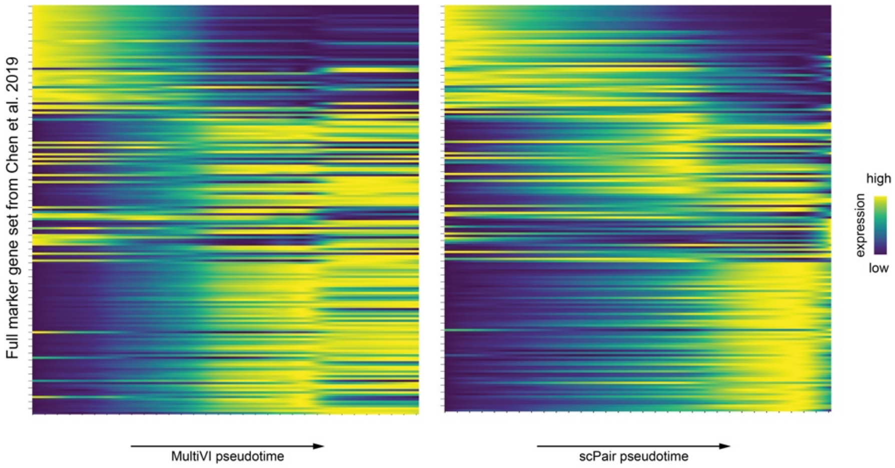

Figure S6

Similar to Figure 4c, but with the full list of marker genes from the original study[4] on the y-axis, with each row representing a gene. Heatmaps display the expression of developmental state markers along pseudotime (x-axis) as inferred from MultiVI (left) and scPair (right) in ATAC space. Source data are provided as a Source Data file.

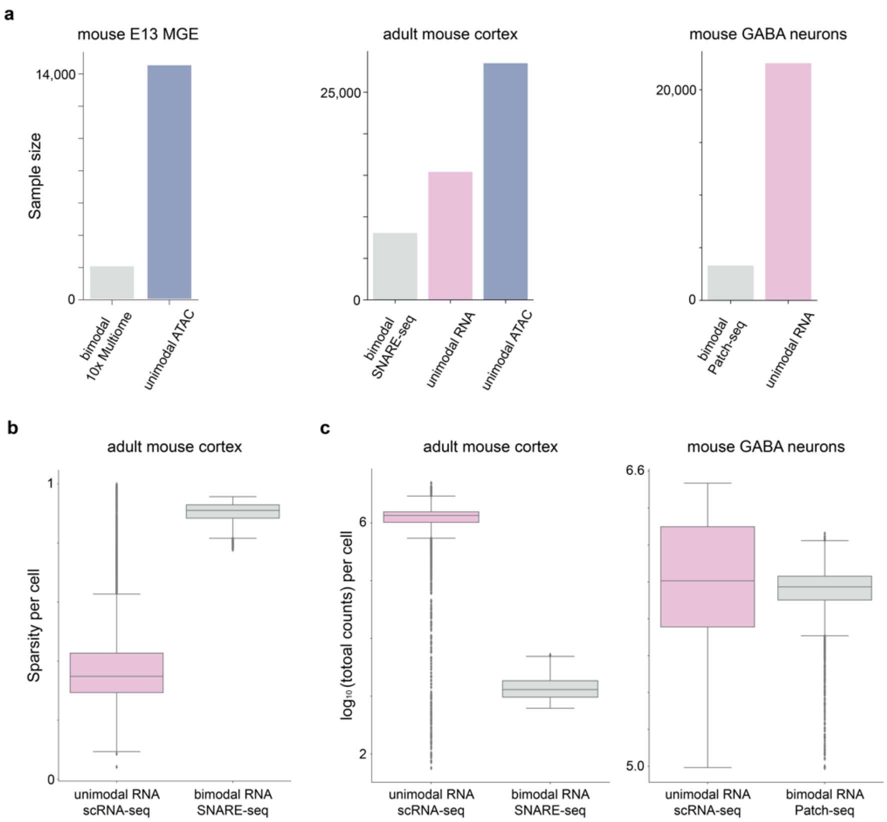

Figure S7

(a) Bar plots show the difference in sample size between bimodal and unimodal datasets[4,15–18] from the same samples analyzed in this study. **(b)** Box plots show the difference in sparsity (percentage of undetected gene features per cell) between bimodal RNA profile of SNARE-seq data[4] and unimodal scRNA-seq dataset from the Allen Brain Institute[16].

(c) Box plots show the difference in sequencing depth (log-transformed total reads per cell) between RNA profiles of bimodal datasets and their corresponding unimodal scRNA-seq datasets from Allen Brain Institute. In the box plots, the minima, maxima, centerline, bounds of box, and whiskers represent minimum value in the data, maximum, median, upper and lower quartiles, and 1.5x interquartile range, respectively. Source data are provided as a Source Data file.

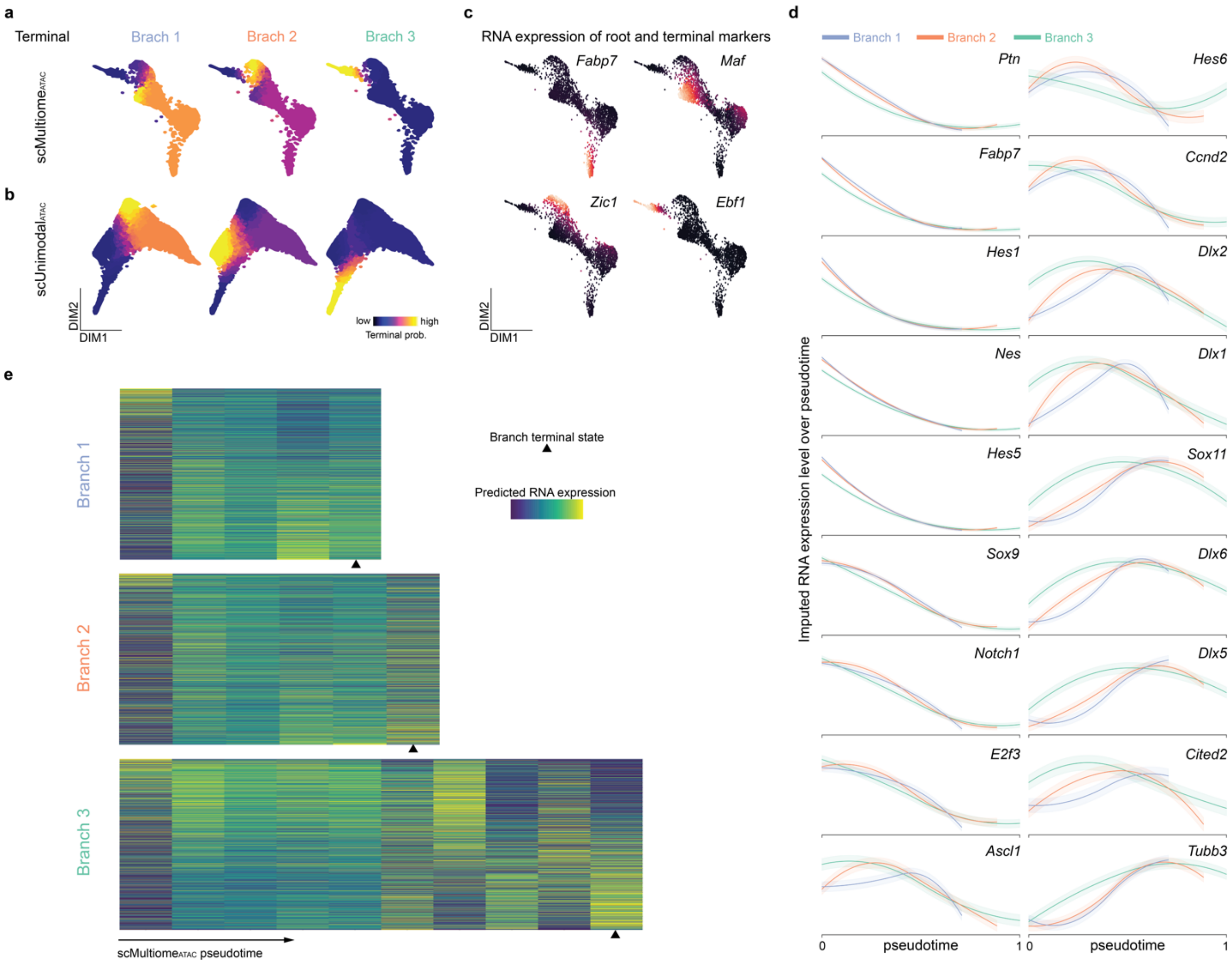

Figure S8

(a-b) UMAP visualizations of ATAC cell states learned by scPair from paired 10x scMultiome (a) and unimodal scATAC-seq (b) data. Cells are colored by branch terminal assignment probabilities based on the inferred trajectories. **(c)** UMAP visualizations of 10x scMultiome data are colored by RNA expression patterns of marker genes that define the trajectory branch starting state (*Fabp7*) and three endpoints (*Maf*, *Zic1*, and *Ebf1*) of mouse postmitotic MGE cells at embryonic day 13 (E13). **(d)** Line plots show the imputed RNA expression from unimodal scATAC-seq data solely for known maturation marker genes from the original study[15] along the inferred pseudotime. **(e)** Heatmaps compare predicted RNA expression changes along inferred pseudotime (x-axis) for each branch using 10x scMultiome ATAC data. Each row of the heatmap represents a gene feature, and each column represents a single 0.05 “pseudotime interval”. In each heatmap, the order of rows from top to bottom is based on the estimated feature-wise pseudotimes (**Methods**) in an ascending manner according to their trajectories that are specific to branches.

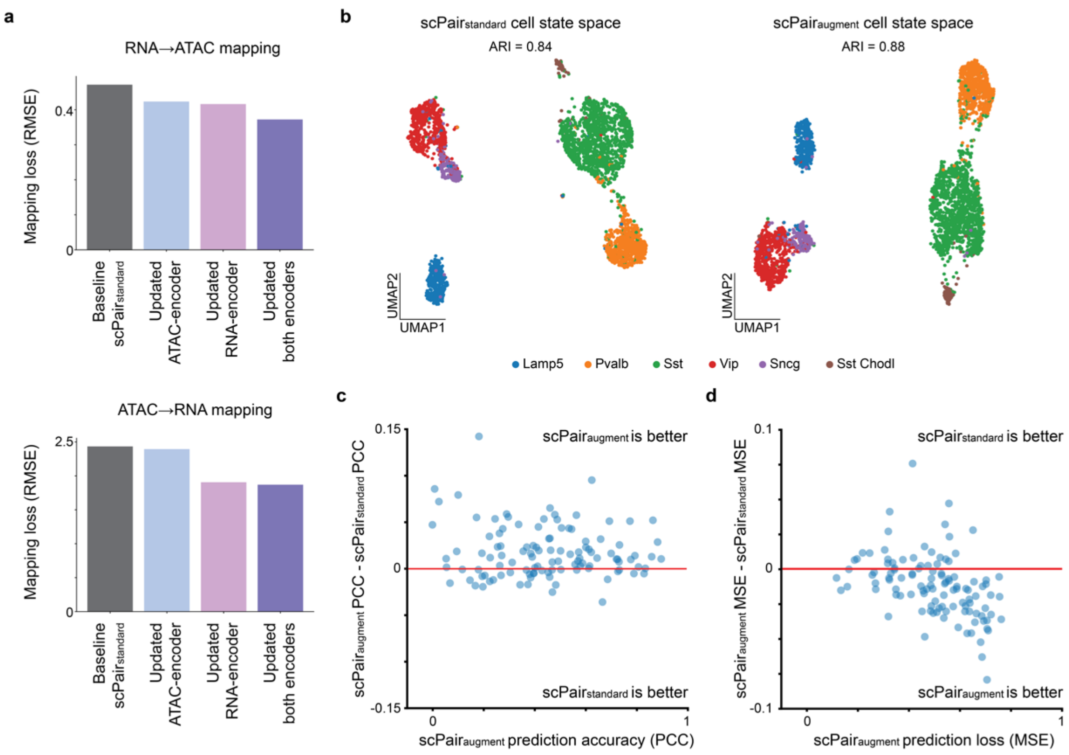

Figure S9

(a) Bar plots quantifying the improvement in cross-modality cell state mapping before and after updating parameters of scPair encoding networks using unimodal datasets (top: RNA → ATAC mapping; bottom: ATAC → RNA mapping). The y-axis shows the Root-Mean-Squared-Error (RMSE) to indicate model performance, with lower values indicating better performance. **(b)** UMAP visualizations of Patch-seq cell states embedded by $\text{scPair}_{\text{standard}}$ (left) and updated $\text{scPair}_{\text{augment}}$ (right) using unimodal datasets. **(c-d)** Prediction of electrophysiological properties from RNA expression profiles improves after incorporating unimodal scRNA-seq data. Scatter plots illustrate prediction accuracy using Pearson correlation coefficients (PCC, c) and prediction loss using mean-squared-error (MSE, d) for individual features after the update (x-axis) with $\text{scPair}_{\text{augment}}$. The y-axis represents the difference between $\text{scPair}_{\text{augment}}$ and $\text{scPair}_{\text{standard}}$. Data points above the y=0 line in (c) and below the y=0 line in (d) indicate improved predictions after incorporating unimodal data. Source data are provided as a Source Data file.

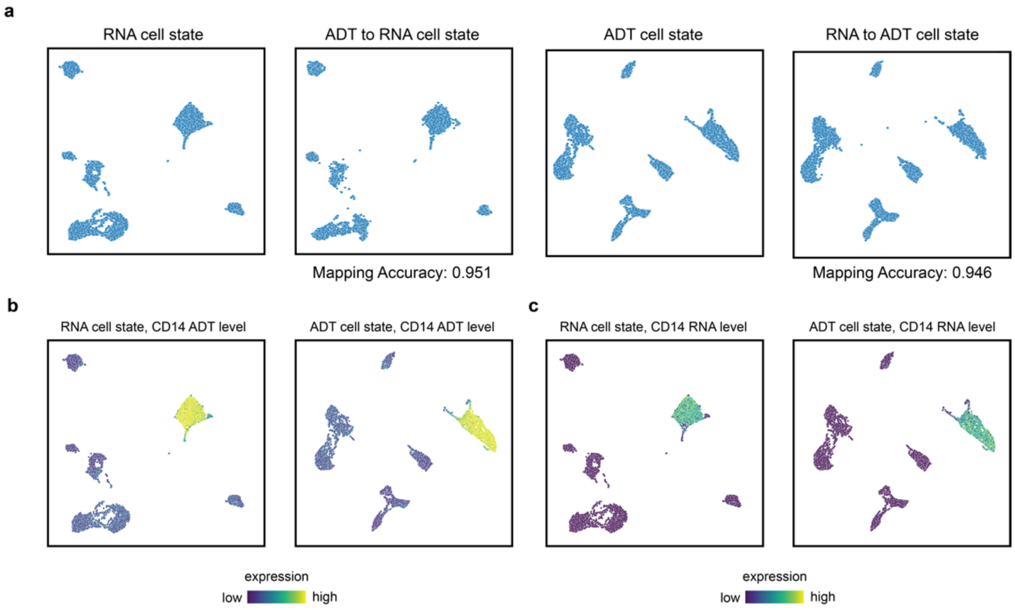

Figure S10

(a) UMAP visualizations of cell state spaces derived from RNA component (left) and Antibody-Derived Tags (ADT, third from left) using scPair on the 10x Genomics PBMC sample CITE-seq (Cellular Indexing of Transcriptomes and Epitopes by Sequencing) data. The corresponding cell states mapped from the alternative modalities are displayed next to each respective visualization. **(b)** UMAP visualizations displaying the expression patterns of CD14 ADT in both cell states. **(c)** UMAP visualizations displaying the expression patterns of CD14 RNA in both cell states. Source data are provided as a Source Data file.

References

1. Cao, J. *et al.* Joint profiling of chromatin accessibility and gene expression in thousands of single cells. *Science* **361**, 1380–1385 (2018).
2. Guyer, R. A. *et al.* Single-cell multiome sequencing clarifies enteric glial diversity and identifies an intraganglionic population poised for neurogenesis. *Cell Reports* **42**, 112194 (2023).
3. Ma, S. *et al.* Chromatin potential identified by shared single cell profiling of RNA and chromatin. *bioRxiv* 2020.06.17.156943 (2020) doi:10.1101/2020.06.17.156943.
4. Chen, S., Lake, B. B. & Zhang, K. High-throughput sequencing of the transcriptome and chromatin accessibility in the same cell. *Nature Biotechnology* **37**, 1452–1457 (2019).
5. Bakken, T. E. *et al.* Comparative cellular analysis of motor cortex in human, marmoset and mouse. *Nature* **598**, 111–119 (2021).
6. Zhang, R., Meng-Papaxanthos, L., Vert, J.-P. & Noble, W. S. Semi-supervised Single-Cell Cross-modality Translation Using Polarbear. in *Research in Computational Molecular Biology* (ed. Pe'er, I.) vol. 13278 20–35 (Springer International Publishing, Cham, 2022).
7. Lopez, R., Regier, J., Cole, M. B., Jordan, M. I. & Yosef, N. Deep generative modeling for single-cell transcriptomics. *Nat. Methods* **15**, 1053–1058 (2018).
8. Ashuach, T., Reidenbach, D. A., Gayoso, A. & Yosef, N. PeakVI: A deep generative model for single-cell chromatin accessibility analysis. *Cell Reports Methods* **2**, 100182 (2022).
9. Ashuach, T., Gabitto, M. I., Jordan, M. I. & Yosef, N. *MultiVI: Deep Generative Model for the Integration of Multi-Modal Data*. http://biorxiv.org/lookup/doi/10.1101/2021.08.20.457057 (2021) doi:10.1101/2021.08.20.457057.
10. Gong, B., Zhou, Y. & Purdom, E. Cobolt: integrative analysis of multimodal single-cell sequencing data. *Genome Biol* **22**, 351 (2021).
11. Stuart, T. *et al.* Comprehensive Integration of Single-Cell Data. *Cell* (2019) doi:10.1016/j.cell.2019.05.031.

12. Stuart, T. & Satija, R. Integrative single-cell analysis. *Nat Rev Genet* **20**, 257–272 (2019).
13. Stuart, T., Srivastava, A., Madad, S., Lareau, C. A. & Satija, R. Single-cell chromatin state analysis with Signac. *Nat Methods* **18**, 1333–1341 (2021).
14. Setty, M. *et al.* Characterization of cell fate probabilities in single-cell data with Palantir. *Nat Biotechnol* **37**, 451–460 (2019).
15. Allaway, K. C. *et al.* Genetic and epigenetic coordination of cortical interneuron development. *Nature* **597**, 693–697 (2021).
16. Yao, Z. *et al.* A high-resolution transcriptomic and spatial atlas of cell types in the whole mouse brain. *Nature* **624**, 317–332 (2023).
17. Fang, R. *et al.* Comprehensive analysis of single cell ATAC-seq data with SnapATAC. *Nat Commun* **12**, 1337 (2021).
18. Gouwens, N. W. *et al.* Integrated Morphoelectric and Transcriptomic Classification of Cortical GABAergic Cells. *Cell* **183**, 935-953.e19 (2020).
19. Ghazanfar, S., Guibentif, C. & Marioni, J. C. Stabilized mosaic single-cell data integration using unshared features. *Nat Biotechnol* **42**, 284–292 (2024).
